# Self-Healable, Transparent, Biodegradable, and Shape Memorable Polyurethanes Derived from Carbon Dioxide-Based Diols

**DOI:** 10.3390/molecules29184364

**Published:** 2024-09-13

**Authors:** Xin Huang, Tingting Zhao, Shuanjin Wang, Dongmei Han, Sheng Huang, Hui Guo, Min Xiao, Yuezhong Meng

**Affiliations:** 1 The Key Laboratory of Low-Carbon Chemistry & Energy Conservation of Guangdong Province, State Key Laboratory of Optoelectronic Materials and Technologies, School of Materials Science and Engineering, Sun Yat-sen University, Guangzhou 510275, China13890773752@163.com (T.Z.);; 2 School of Chemical Engineering and Technology, Sun Yat-sen University, Zhuhai 519000, China; handongm@mail.sysu.edu.cn (D.H.);; 3 Institute of Chemistry, Henan Provincial Academy of Sciences, Zhengzhou 450000, China; 4 College of Chemistry, Zhengzhou University, Zhengzhou 450001, China

**Keywords:** CO_2_, poly(polycarbonate) diol, thermoplastic polyurethanes

## Abstract

A series of CO_2_-based thermoplastic polyurethanes (TPUs) were prepared using CO_2_-based poly(polycarbonate) diol (PPCDL), 4,4′-methylenebis (cyclohexyl isocyanate) (HMDI), and polypropylene glycol (PPG and 1,4-butanediol (BDO) as the raw materials. The mechanical, thermal, optical, and barrier properties shape memory behaviors, while biocompatibility and degradation behaviors of the CO_2_-based TPUs are also systematically investigated. All the synthesized TPUs are highly transparent amorphous polymers, with one glass transition temperature at ~15–45 °C varying with hard segment content and soft segment composition. When PPG is incorporated into the soft segments, the resultant TPUs exhibit excellent self-healing and shape memory performances with the average shape fixity ratio and shape recovery ratio as high as 98.9% and 88.3%, respectively. Furthermore, the CO_2_-based TPUs also show superior water vapor permeability resistance, good biocompatibility, and good biodegradation properties, demonstrating their pretty competitive potential in the polyurethane industry applications.

## 1. Introduction

Polyurethane (PU) is a common and versatile polymeric material that has become an integral part of our daily lives since its discovery by Otto Bayer in 1937. Thermoplastic polyurethanes (TPUs) have drawn significant attention in the field of PUs due to good resistance to impact, abrasion, chemicals, and weather, as well as their recyclability and ability to undergo thermal processing methods such as extrusion, blow, injection molding, and thermoforming [1]. Hence, TPUs have possessed extensive applications in various industries such as packaging, photovoltaics, sports equipment, construction, and thermal insulation materials [2,3].

TPUs produced from conventional polyester polyols exhibit good mechanical properties, but their usage is limited due to poor hydrolysis resistance [4,5,6]. On the other hand, TPUs produced from polyether polyols generally have good hydrolysis resistance but tend to have poor oxidation resistance and mechanical properties [7,8]. Poly(carbonate urethane)s (PCUs) prepared using polycarbonate diols (PCDLs) as the soft segment offer numerous advantages over PUs synthesized from conventional polyether and polyester diols, including exceptional mechanical properties, hydrolysis resistance, abrasive resistance, oil resistance, chemical resistance, and excellent biocompatibility [9,10,11,12]. As a result, the demand for PCDLs as a feedstock for PCUs has been increasing in recent years.

Various methods have been used to prepare PCDLs, including the reaction of diols with phosgene or phosgene derivatives [13], ring-opening polymerization of cyclic carbonates [14], transesterification of dimethyl carbonate with micromolecule diols [15], and alcoholysis of high-molecular-weight poly(propylene carbonate) [16]. The increasing focus on environmental protection and sustainable development has led to the recognition of CO_2_ as a crucial raw material for chemical and polymer syntheses [17,18,19], of which the direct utilization of CO_2_ in the production of degradable low-molecular-weight polycarbonate polyols or oligo(carbonate-ether) polyols through epoxides/CO_2_ copolymerization has gained significant attention from both academic research and industrial productions over the past few decades [13,14,20,21,22,23]. This method offers an eco-friendly alternative resource for producing polyols, as CO_2_ is directly incorporated into the carbonate units of polyols via copolymerization.

The progress in the synthesis of CO_2_-based PCDLs boosts the development of CO_2_-based polyurethanes, which offers an effective way to reduce fossil energy consumption [24]. Wang et al. investigated the properties of waterborne PUs derived from oligo(carbonate-ether) diol, which exhibited better hydrolysis and oxidation resistances compared to PUs from polyether and polyester polyols [9]. Hong et al. successfully prepared a TPU with extraordinary shape memory properties using CO_2_-based polyols, exhibiting shape fixity and shape recovery values close to 100% [25]. Lee et al. proposed a preparative method for a flame-retarding TPU whose glass transition temperature can be tuned between 40–60 °C using CO_2_-based poly(propylene carbonate)-diols [26].

Despite these significant progresses, the utilization of CO_2_-based polyols in the preparation of PUs is still limited. The characteristics of PUs with CO_2_-based polyols need to be thoroughly evaluated to determine their potential applications.

In our previous work, we have successfully synthesized a series of poly(propylene carbonate)-diols or poly(carbonate-ester) diols with precisely controllable molecular weight from CO_2_ in the presence of BDO (1–4 butanediol) [27,28]. We have also demonstrated that the sufficient primary hydroxyl group is capped with the chain end to ensure reactivity in subsequent reactions. The incorporation of CO_2_-based polycarbonate as soft segment into TPUs is expected to endow them with good mechanical performance, hydrolytic stability, UV aging resistance, and gas-barrier properties, as well as biodegradation. This addresses the limitations of TPUs produced from conventional polyester and polyether polyols [29,30,31,32].

Herein, the utilization of CO_2_-based poly(propylene carbonate)-diols (PPCDL) and polyether polyols (PPG) as soft segments for preparing TPUs is explored. The mechanical properties and shape memory behavior, thermal performance, optical and barrier properties, biocompatibility, and degradation behavior of the CO_2_-based TPUs are systematically investigated. This study addressed the challenge of carbon dioxide fixation by effectively utilizing CO_2_-based polyols in polyurethane synthesis, which offers a sustainable feedstock as the substitution of petro-based raw materials for the PU industry.

## 2. Results and Discussion

### 2.1. Structure of CO_2_-Based PPCDL

The chemical structure of a typical CO_2_-based PPCDL was analyzed by means of ^1^H NMR and matrix-assisted laser-desorption ionization time-of-flight mass spectroscopy (MALDI-TOF), as illustrated in Figure S1a,c. The signals at 4.8–5.0 ppm, 3.9–4.3 ppm, and 1.3–1.5 ppm in Figure S1a depict the protons of CH, CH_2_, and CH_3_ groups of the carbonate units, respectively [16]. A minor amount of ether unit is noticed in the polyol, evidenced by the low intensity of the signal at 3.3–3.6 ppm in ^1^H NMR [33]. The content of the carbonate unit in the PPCDL is calculated to be 93 mol%.

The *M*_n_ of the PPCDL is determined to be 1950 g/mol with a narrow PDI value according to the GPC curve shown in Figure S1b. Meanwhile, the *M*_n_ value of PPCDL determined by MALDI TOF (Figure S1c) is merely 1700 g/mol, which is lower than that measured by GPC. This difference may be attributed to linear polystyrene as a standard sample in the GPC analyses. MALDI TOF mass spectrum shows that there exist three main groups of molecular ions with the discrepancy of the number of ether linkages in each group. The interval between peaks within each group corresponds to the molecular weight of the PPC repeating unit, and all the molecules are hydroxyl terminated.

### 2.2. Structure of CO_2_-Based TPU

**Scheme 1:**
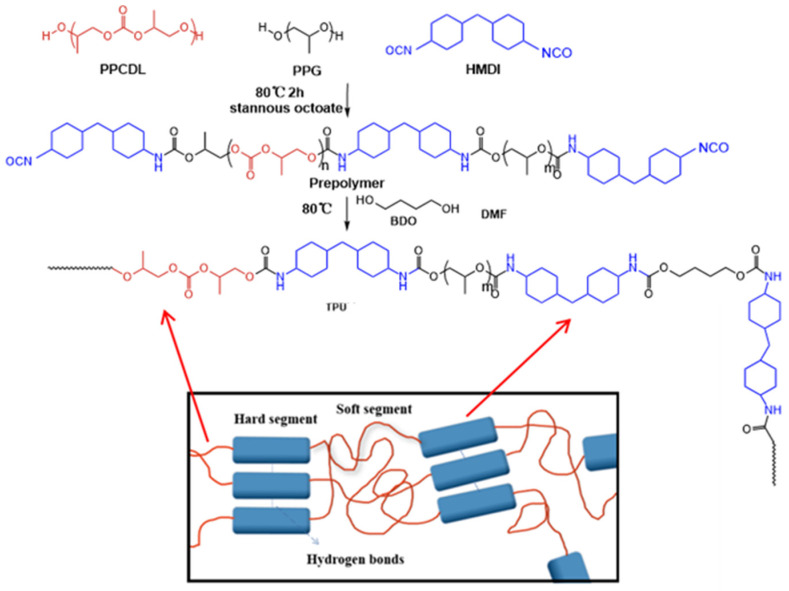
Schematic representation of the preparation of TPUs with the mixture of PPCDL and PPG.

**Figure 1:**
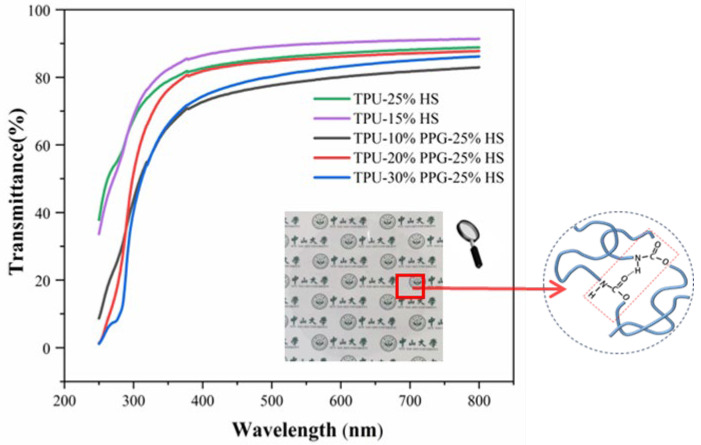
Transmittance and physical pictures of the TPUs.

To examine the effects of different polyol compositions on the properties of thermoplastic polyurethanes (TPUs), we synthesized TPUs with a hard segment content of 25% using a mixture of polyols comprising PPCDL and conventional polypropylene glycol (PPG) with varying ratios ([PPG]/[PPCDL] = 10%, 20%, 30% weight ratio), designed as TPU-10% PPG-25% HS, TPU-20% PPG-25%, and HS TPU-30% PPG-25% HS in turn. TPUs with a single PPCDL soft segment but different hard segment content (TPU-15% HS and TPU-25% HS) were also synthesized for comparison. All the TPUs were prepared through two-step reactions in the presence of the catalyst [30,32]. The recipes for the preparation of TPUs are presented in Table 1. Scheme 1 presents a detailed schematic diagram of the reaction pathways involved in the preparation of TPUs, emphasizing the critical steps necessary for successful TPU synthesis. To prevent yellowing and degradation of the sample due to exposure to UV light, aliphatic diisocyanate (HMDI; 4,4′-methylenebis (cyclohexyl isocyanate)) was used [34]. The content of the hard segment in the TPUs was consistently regulated, and the *M*_n_ values were carefully adjusted to ensure similarity among all TPUs (Figure S2). Notably, all the obtained TPUs are transparent and colorless (Figure 1).

**Figure 2:**
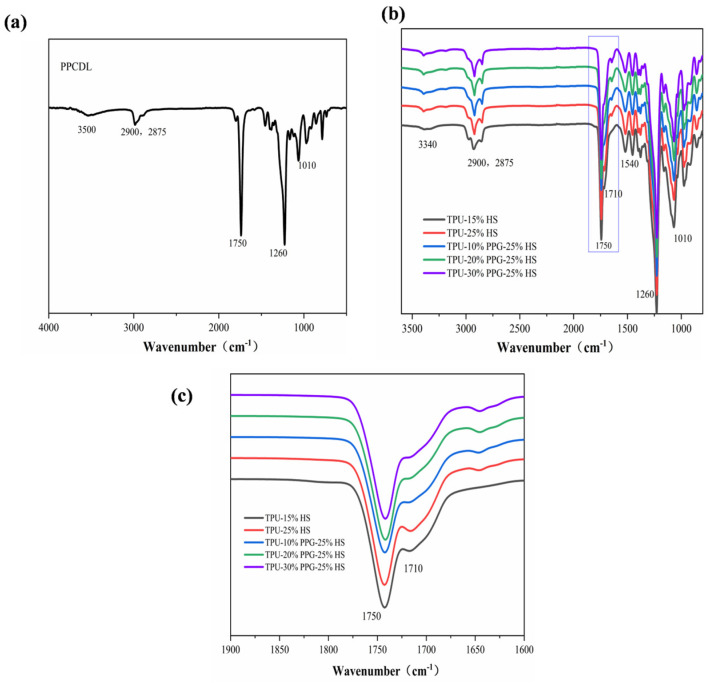
FT-IR spectra of (**a**) PPCDL, (**b**) TPU films, and (**c**) TPU films with 1600 to 1900 cm^−1^ zoomed.

The FT-IR of the PPCDL and the corresponding TPU are shown in Figure 2a,b, respectively. The IR spectrum of PPCDL shows typical strong stretching-vibration absorption bands of -C=O at 1750 cm^−1^ and an absorption peak of -OH group at 3500 cm^−1^ [20]. In the IR spectrum of TPUs, the peaks of –OH disappear, while new peaks at 3340 cm^−1^ and 1540 cm^−1^ belonging to the -NH group and -NH-CO- appear, which indicates the successful formation of the urethane group [4,25,30,32]. ^1^H NMR (Figure S3) shows the resonance at 7.01–7.05 ppm corresponding to the urethane -NH-proton, also indicating the successful formation of TPU [25]. It is well known that the hydrogen-bonded C=O stretching vibration appears at a lower wavenumber as compared with the free C=O stretching vibration [35,36]. From the zoomed FT-IR results of polyurethane in the range of 1600 to 1900 cm^−1^ (Figure 2c), we can see that all the TPUs show a shoulder hydrogen-bonded C=O peak (1710 cm^−1^) accompanied by the free C=O peak (1750 cm^−1^), indicating the formation of hydrogen bonds between C=O of the soft segment and N-H of the hard segment.

### 2.3. Properties of Synthesized TPU

#### 2.3.1. Thermal Properties

**Figure 3:**
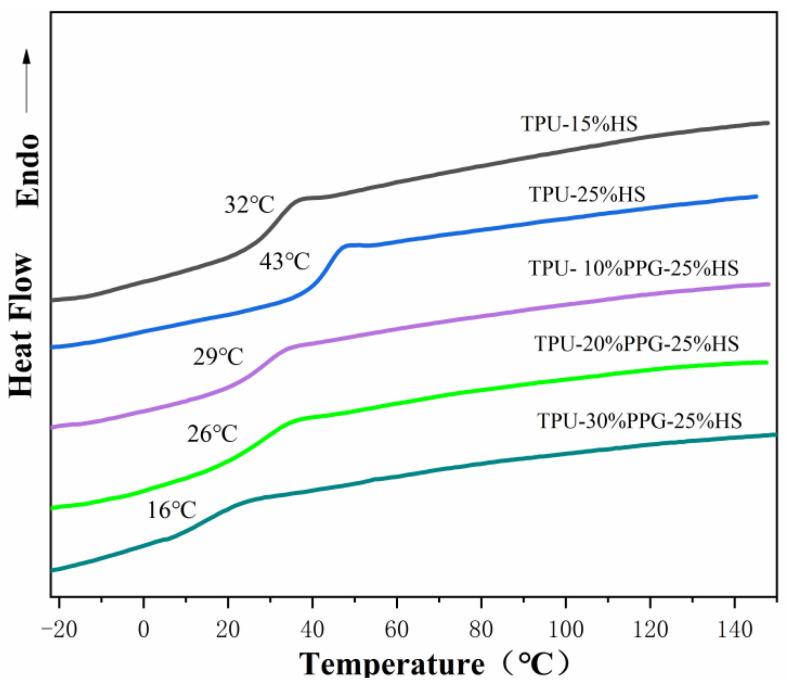
DSC of TPUs (second heating run).

The PPCDL exhibited a *T*_g_ of −12 °C (Figure S4), while the TPUs synthesized from the PPCDL demonstrate higher *T*_g_s (43 °C and 32 °C), increasing with the increase of the hard segment content, as shown in Figure 3. The increase of *T*_g_ of TPUs as compared with PPCDL is due to the restriction of the movement of the soft segment by hydrogen-bond interaction between C=O of the soft segment and N-H of the hard segment [10,37]. All the TPUs synthesized from the mixture diols of PPG and PPCDL show lower *T*_g_s as compared with those with PPCDL as a single soft segment, and a decrease in *T*_g_ values from 29 °C to 16 °C is observed when the proportion of PPG in the mixed polyols increased from 10% to 30% (Figure 3 and Table 2). This result could be attributed to the greater flexibility of the PPG compared to PPCDL.

Figure S4 presents the WAXD spectra of TPU samples with different compositions. A broad diffraction peak is observed at around 19° for all five samples, demonstrating the more amorphous nature of the TPUs.

**Figure 4:**
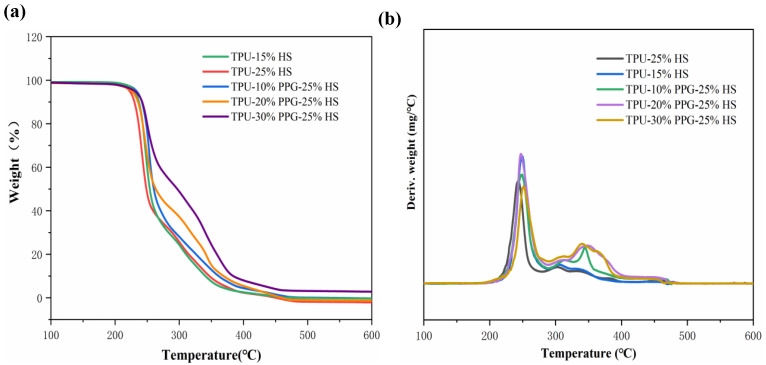
(**a**) TGA curves and (**b**) weight-loss derivative curves of TPUs.

TGA curves along with the derivative curves are presented in Figure 4, and related data are summarized in Table 2. It is apparent that all TPUs exhibit reasonable thermal stability, with a 5% weight loss temperature (*T*_5%_) ranging from 220 to 231 °C. DTG curves of PPCDL-TPUs show two distinct weight losses, indicating a two-step degradation process. The initial step occurs in the temperature range of 220–270 °C, where the carbonate units undergo decomposition. The second step occurring between 270–350 °C is attributed to the decomposition of TPUs’ urethane linkage [11,38,39]. TPUs containing both PPCDL and PPG soft segments show three-step degradation processes, which are the degradation of the PPCDL soft segment, urethane linkage, and PPG soft segment. It is apparent that the thermal stability of the PPCDL/PPG-based TPU films is higher than that of PPCDL -TPU films due to the incorporation of polyether blocks.

#### 2.3.2. Mechanical and Adhesive Properties

**Figure 5:**
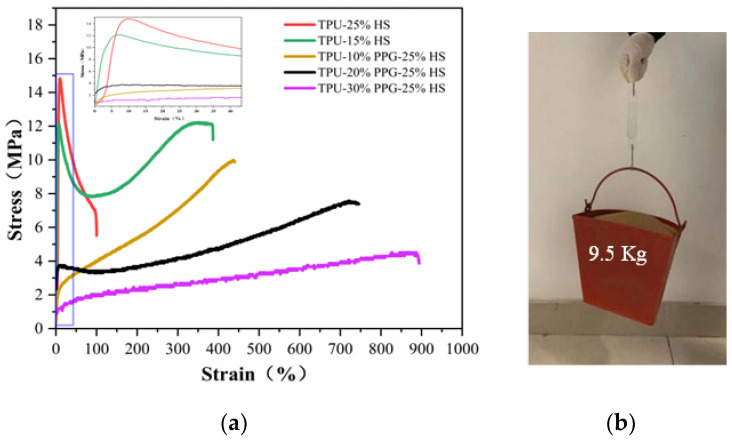
(**a**) Tensile stress–strain curves of TPUs and (**b**) adhesive property.

Figure 5a depicts the stress–strain curves of the TPUs, accompanied by a listing of their corresponding mechanical properties in Table 2. TPUs consisting solely of PPCDL exhibit characteristics of plastics and are found to possess a relatively lower level of elongation at break (98.5%) when the hard segment is up to 25%. Decreasing the hard segment content to 15% results in a decrease of tensile strength to 12.1 MPa, with a dramatic increase of the elongation at break to 384%. In comparison to PPCDL-TPU with a hard segment content of 25%, the PPG- and PPCDL-based TPUs with similar hard segment contents show lower tensile strength and higher elongation at the break due to the introduction of higher flexible PPGs as the second soft segment. With the content of PPG in the mixed polyols increasing from 10% to 30%, the elongation at break values of the TPUs increased from 438% to 886% (Figure S6 shows the digital photo of deformation during the tensile process), while the tensile strength of TPU-30%PPG-25%HS can maintain only 4.52 MPa, demonstrating good elastomeric properties. These might be ascribed to abundant hydrogen bonds between carbonate in the soft segment and urethane groups in the hard segment. Moreover, the TPUs show extraordinary adhesive properties, as shown in Figure 5b. Two glasses specimens adhered by TPU (50 mg, 20 mm wide, 30 mm long) can successfully lift a weight of 9.5 kg, which is 190,000 times greater than their own weight. The superb adhesion to glass may result from the formation of hydrogen bonding with the hydroxyl groups on the silicate surface.

#### 2.3.3. Shape Memorable and Self-Healable Properties

**Figure 6:**
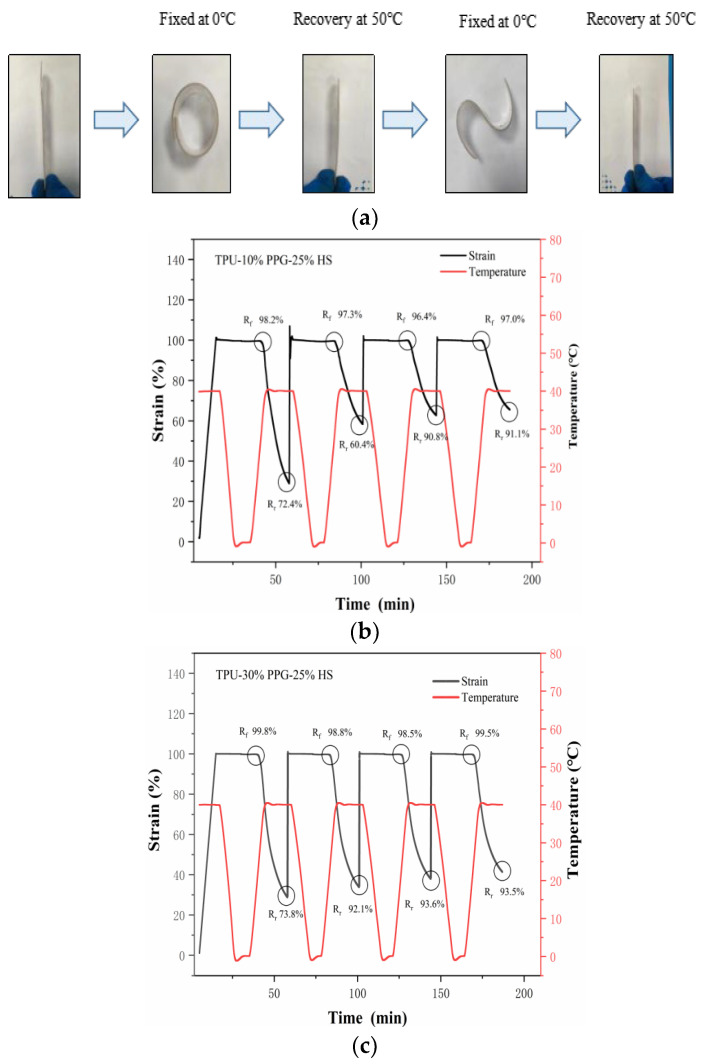
(**a**) Shape memory performance photos of TPU-30% PPG-25% HS, strain–time–temperature diagram of (**b**) TPU-10% PPG-25% HS and (**c**) TPU-30% PPG-25% HS with a thermally induced shape memory effect.

Because of the soft and hard multiblock structure of TPU, the hard segments form physical cross-links through hydrogen bonding, which plays the role of a fixed phase. The soft segments can be reversibly softened and cured above and below their glass transition temperatures. Therefore, the synthesized TPUs exhibit good shape memory properties. As shown in Figure 6a and Figure S7, the linear TPU-30%PPG-25%HS and TPU-10%PPG-25%HS were bent into different shapes, which was then cooled to 0 °C to retain the deformation. Upon heating to 50 °C, the shape was completely restored to the linear shape.

The shape memory properties of TPU-10%PPG-25%HS and TPU-30%PPG-25%HS were further investigated by thermo–mechanical cycling tests. As exhibited in Figure 6b, both samples show good shape fixation ability and shape recovery ability; especially after several thermo–mechanical cycles, the overall shape recovery rate tends to be stable. The reason is that disentanglement can occur when the molecular chain is loaded at a temperature higher than *T*_g_. After lowering the temperature to 0 °C and unloading, the molecular chain will be entangled together again in the process of heating. A series of thermo–mechanical cycling led to lower residual stresses and plastic deformation within the chain and improved overall molecular chain stability, thereby promoting more consistent response parameters throughout cycling [25,40]. Shape recovery performance strengthens with increasing PPG content, as depicted in Table 3; TPU-30%PPG-25%HS has an average R_f_ value of 98.9% and Rr value of 88.3%, which exceeds that of TPU-10%PPG-25%HS (R_f_ = 97.6% and Rr = 78.7%). This is attributed to the fact that the higher PPG content led to softer segments and enhanced molecular chain mobility, ultimately facilitating deformation recovery [41].

**Figure 7:**
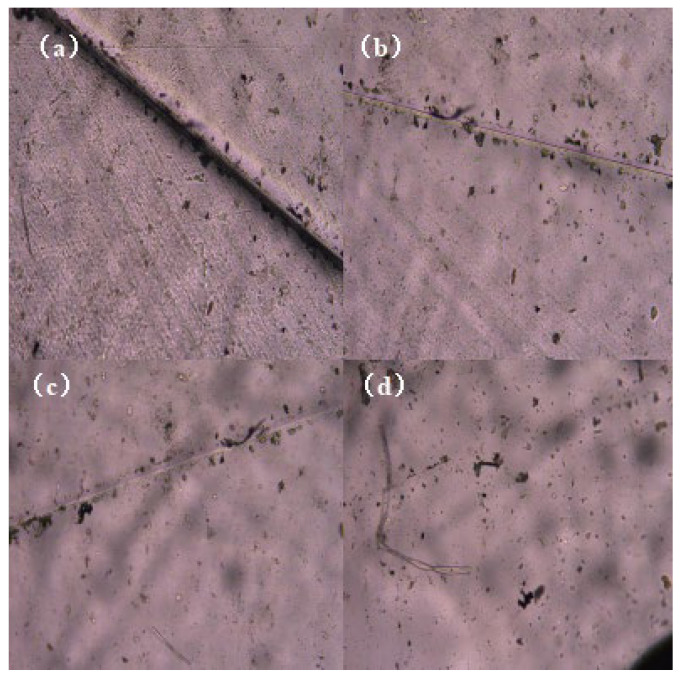
Evolution of the cutting gap of the TPU-20% PPG-25%HS being healed at 60 °C for (**a**) 0 min (**b**) 10 min (**c**) 20 min and (**d**) 60 min.

More interestingly, optical microscopic images (Figure 7) demonstrate that TPU-20% PPG-25%HS is healable at 60 °C, spontaneously repairing the cut gap after 60 min. At first, the gap is a black line under the microscope. Over time, the black line fades, illustrating that the gap gradually disappears. The process is considered to mainly be attributed to the reorganization of intramolecular and intermolecular hydrogen bonds.

#### 2.3.4. Water Vapor Permeability Resistance and Biocompatibility

To measure the moisture permeability of the TPUs, water vapour transmission tests were performed. Table S1 shows the water vapour transmission rate (WVTR) and water vapour transmission coefficient (WVP) data of the TPUs. Apparently, the PPCDL-based TPUs exhibit excellent water vapour-barrier properties with a WVP as low as 170 g·μm·m^−2^·day^−1^, which is attributed to the high gas-barrier property of PPC soft segments, as well as the further tightening of the molecular chain arrangement by the hydrogen-bond-rich urethane hard segment linkages [42]. Increasing the hard segments from 15% to 25% resulted in a slight improvement in the water vapour-barrier performance of the TPU membrane. However, in contrast to TPUs that solely consist of PPCDL, PPCDL-PPG-TPU showed significantly elevated moisture permeability. As the PPG content in TPU films increases from 10% to 30%, the WVP of TPU films exhibits a gradual rise from 183 to 299 g·μm·m^−2^·day^−1^. The occurrence of this phenomenon may be due to good hydrophilicity of the PPG segment. The increased proportion of hydrophilic chain segments in the molecular chain results in improved hydrophilicity and the ability to capture water molecules, ultimately leading to an enhanced moisture permeability of the films [43]. 

**Figure 8:**
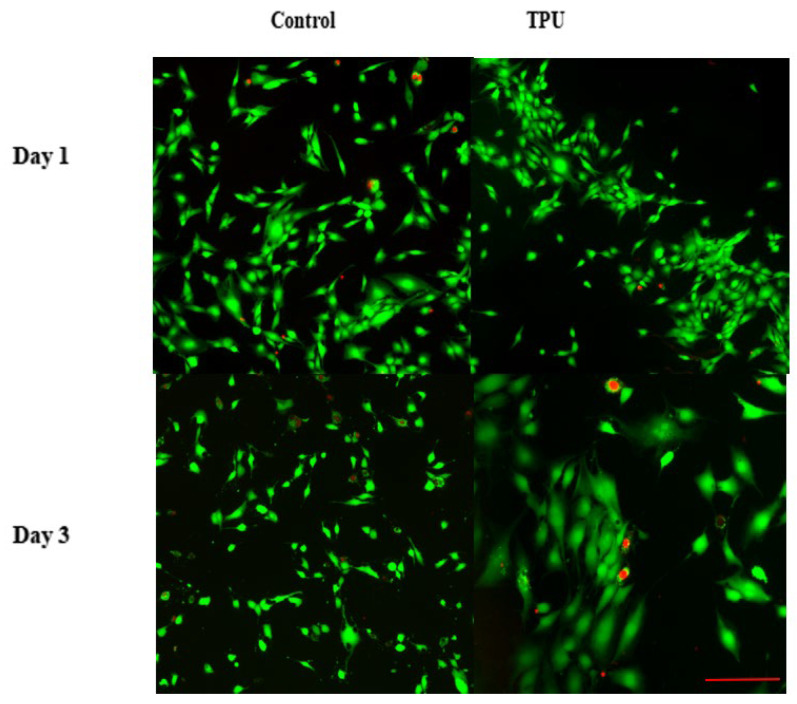
Morphology of 3T3 adherent to TPU surfaces after 1, 3 days culture. TPU-20%PPG-25% HS was used as an example.

In order to assess the in vitro biocompatibility of the TPU elastomer, cytotoxicity testing was conducted. Specifically, the selected TPU elastomer substrates were cultured with the widely used 3T3 mouse fibroblast cell line. As depicted in Figure 8, fluorescence microscopy images of fluorescein diacetate/GelRed TM-stained 3T3 cells growing on the elastomer and glass control substrates were captured after several days of cell culture. Notably, the TPU substrate exhibited excellent cell adhesion and proliferation of 3T3 cells, which proved that CO_2_-based-TPU membranes had no toxic effect on cells, thereby indicating their biocompatibility and potential applicability in biomedical fields.

#### 2.3.5. Biodegradability

**Figure 9:**
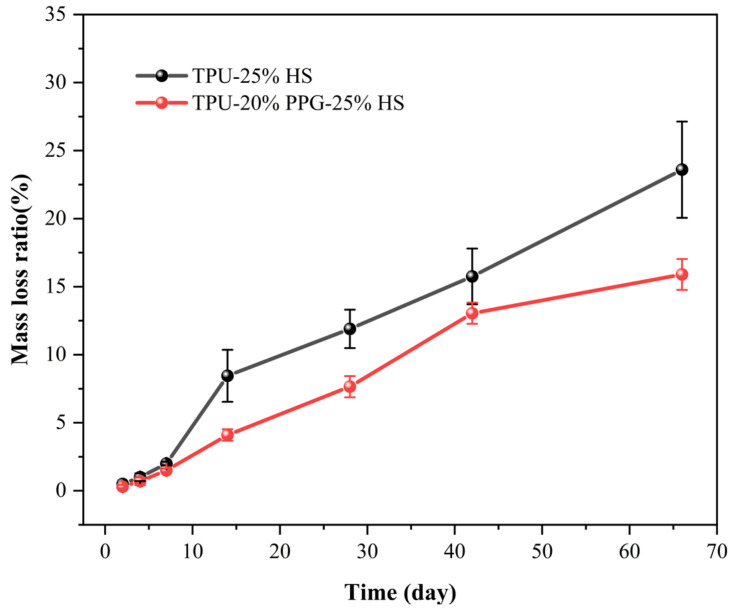
Quality changes of TPU enzymatic hydrolysis.

In order to evaluate the biodegradation performance of the CO_2_-based TPUs, TPU-25%HS and TPU-20%PPG-25%HS were selected to compare the enzymatic degradation. Enzymolysis experiments were conducted in PBS buffer solution spiked with antarctic pseudohyphae enzyme (CALB) at 45 °C. The test results are shown in Table 4 and Figure 9. The mass loss of the samples increased with the extension of enzymolysis time. After 66 days of enzymatic hydrolysis, the TPUs exhibited localized small pores and cracks on the sample surface, as shown in Figure S8. The mass loss of TPU-25%HS reached 23.6 wt%, and the *M*_n_ was reduced from 38,000 g/mol to only 8500 g/mol, whereas the mass loss of TPU-20%PPG-25%HS PPG was 15.9 wt%, implying that it has better enzymatic stability.

**Figure 10:**
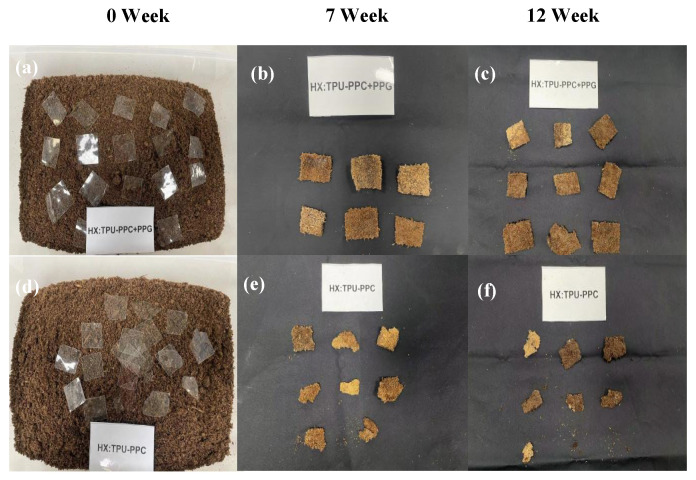
Images of TPU-20%PPG-25%HS (**a**–**c**) and TPU-25% HS (**d**–**f**) films before and after composting for 7 and 12 weeks.

Except for enzymolysis, a compost degradation experiment was also taken to evaluate the biodegradability of the TPUs. As shown in Figure 10d–f, the TPU-25%HS samples with only PPCDL as soft segments incubated in the compost were characterized by the appearance of cracks and eventually breaking into smaller pieces. In contrast, TPU-20%PPG-25%HS only showed some cracks, which reflects the relatively lower biodegradation rate under the composting condition (Figure 10a–c). In addition, the decrease of *M*_n_ of the TPUs agrees with the images recorded in the composting degradation test. Specifically, *M*_n_ of TPU-25%HS decreased from 38,000 to 9900 g/mol, while *M*_n_ declined from 36,000 to 28,000 g/mol for the TPU-20% PPG-25% HS (Figure S9 and Table S2), demonstrating a better biodegradability of the soft segment consisting of a single PPCDL.

## 3. Materials and Methods

### 3.1. Material

CO_2_ (99.999%) was purchased from Guangqi gas Co. (Haizhu District, Guangzhou, China). Propylene oxide (PO) was purchased from Energy Chemical (Qingpu District, Shanghai, China) and was refluxed for 24 h by CaH_2_ and distilled under N_2_ atmosphere. Then, 1,4-Butanediol (BDO) was obtained from Aladdin Co. (Huangpu District, Guangzhou, China). and was dehydrated with 3 Å molecular sieve to ensure the water content was less than 200 ppm. Triethyl boron (TEB 1M in THF) and Polypropylene glycol (PPG) were also purchased from Aladdin Co. (Huangpu District, Guangzhou, China). Bis(triphenylphosphine)iminium chloride (PPNCl) was obtained from TCI (Pudong District, Shanghai, China). Anhydrous N, N-Dimethylformamide (DMF), stannous octoate, and 4,4′-methylenebis (cyclohexyl isocyanate) (HMDI) were purchased from Macklin Co. (Pudong District, Shanghai, China).

### 3.2. Preparation of PPCDLs

In a glove box free of oxygen and water, PPNCl (0.233 g, 0.413 mmol), TEB (3.72 mL, 3.72 mmol), PO (60 g, 1.03 mol), and BDO (2.23 g, 24.8 mmol) were added in a 100 mL stainless-steel reactor equipped with a magnetic stirring. The gas line was cleaned several times to guarantee it was free of moisture before CO_2_ was pressurized into the mixture. The reaction was conducted at 45 °C under 1 MPa CO_2_ for 24 h. After copolymerization, the reactor was cooled to room temperature, and CO_2_ was slowly vented by opening the outlet valve. The product was poured into water, washed for three times, and subsequently dried at 80 °C in vacuum oven until it became transparent.

### 3.3. Preparation of PPCDL and PPG-Based Thermoplastic Polyurethanes

The TPUs were synthesized by a two-step reaction. Firstly, isocyanate-terminated PU prepolymers were prepared by the reaction between the PPCDL/PPG and an excess of HMDI. Calculated amounts of diols were put into a three-necked flask equipped with a vacuum resource, a thermometer, and an N_2_ inlet. After adding HMDI, the temperature gradually elevated to 80 °C, and the reaction persisted for 2.5 h. Then, calculated amount of BDO was slowly added to the system for chain extension. Meanwhile, some solvents (DMF) and catalysts (stannous octoate) were added into the system. The reaction continued at 80 °C for another 2 h. Upon cooling to room temperature, the mixture was slowly poured into methanol, and the product was sedimented and dried under vacuum at 80 °C for 24 h.

### 3.4. Measurement

Hydroxyl values of the PPCDLs were determined by titration according to GB/T12008.3-2009 [44]. Fourier transform infrared spectra (FTIR) were performed with a PerkinElmer Sepctrum100 System (Shelton, CT, USA). ^1^H NMR spectra were recorded on a Bruker Advanced III 400 spectrometer (Billerica, MA, USA) using CDCl_3_ and DMSO as solvents. The *M*_n_ and polydispersity index (PDI) of the PPCDL were tested by GPC (Waters instrument) with polystyrene as standard and THF as eluents. MALDI-TOF spectra were recorded on a Bruker ultrafleX MALDI-TOF spectrometer (Billerica, MA, USA), with 2,5-dihydroxybenzoic acid as the matrix and methyl alcohol as solvent. The glass transition temperatures (Tgs) were determined by differential scanning calorimeter (DSC, Netzsch Model 204, FB, Selb, Germany) with a heating and cooling rate of 10 °C/min from −50–150 °C under nitrogen atmosphere. The Tg values were read from the second heating curves. XRD analysis was performed using an Empyrean over the scanning range between 5° and 60° at a scanning rate of 8°/min. TGA and DTG curves were acquired on a PerkinElmer Pyris Diamond TG/DTA analyzer (Shelton, CT, USA) at a scanning speed of 10 °C min^−1^ (from room temperature to 600 °C) under the nitrogen atmosphere. Tensile properties were characterized on a universal testing machine (Labthink Co., Shandong, China (C610M)) at 25 °C and relative humidity of 50% ± 5% according to GB/T 1040.2-2006 [45] with an extension speed of 100 mm/min. The dimension of the sample was 25 × 4 × 1 mm^3^ dumbbell specimen. The data were reported by the average of three measurements. Shore A hardness test of TPUs was characterized by a SHSIWI sclerometer (Shanghai, China). The sample was a round specimen (diameter = 25 mm, thickness = 2 mm). At least three specimens overlapped for testing. The UV–Vis spectra of the samples were recorded following a wavelength range of 200 to 800 nm using a UV–Vis spectrophotometer (Lambda 900, PerkinElmer, Shelton, CT, USA). The microscope (CEWEI, Shenzhen, China) was used to observe the repair of scratches in polyurethane elastomer films.

The water vapor transmission rate (WVTR) measurements were performed at a temperature of 23 °C and a relative humidity of 85%. The WVTRs were determined using the infrared detection sensor method as specified in ISO 15106-2 [46] and a WVTR analyzer (Permatran-W Model 3/61, Mocon Inc., Brooklyn Park, MN, USA). Water vapor-permeation coefficient (WVP) was calculated according to the following Equation (1):

WVP = WVTR × d(1)

WVP refers to the water vapor-permeability coefficient (g·μm/(m^2^·day)), while WVTR is the water vapor-transmission rate (g/(m^2^·day)), and d refers to the thickness of films (μm).

Data were collected using DMA (242D, NETZSCH, Selb, Germany). The shape memory properties of TPUs were assessed by following established procedures. The sample was clamped and heated to 40 °C at a rate of 5 °C·min^−1^ and held for 10 min. The resulting strain was recorded as ε_0_. Next, a suitable force was applied, and the sample was cooled to 0 °C at a rate of 5 °C min^−1^. During the process, the maximum strain of 100% under tension was recorded as ε_1_. After removing the external force, the strain at that moment was recorded as ε_2_. The sample was then heated to 40 °C at a rate of 5 °C·min^−1^ and held for 15 min, during which the shape recovered. The strain at this time was recorded as ε_3_. Steps (2) to (3) were repeated four times. The shape fixity (R_f_) and shape recovery (R_r_) ratios were calculated using the following equations:

R_f_ (%) = (ε_2_ − ε_0_)/(ε_1_ − ε_0_) × 100 (2)

R_r_ (%) = (ε_2_ − ε_3_)/(ε_2_ − ε_0_) × 100 (3)

In order to evaluate the cytocompatibility of the TPU obtained, NIH 3T3 fibroblast cells were employed. This cell line is widely accepted as a reference for cytocompatibility method due to its easy maintenance in culture and sensitivity to toxicity. The cells are cultured in 96-well plates at a density of 5000 cells/cm^2^ using DMEM medium supplemented with 10% calf bovine serum and 4.5 g/L glucose (Mediatech, Manassas, VA, USA). The cultures were maintained at 37 °C with 95% humidity and 5% CO_2_. As a control, we also cultured cells without TPU. Microscopy images (Nikon-Ti_2_, Tokyo, Japan) were captured at 24 and 72 h after cell seeding to observe changes in morphology and cell proliferation, with green fluorescence indicating living cells and red fluorescence indicating dead cells.

The enzymolysis of polyurethanes in vitro was evaluated by pressing TPU films and weighing them. The samples were then placed in 25 mL vials with antarctic pseudofilamentous yeast enzyme (CALB) and PBS (pH = 7.2–7.4), which is refreshed weekly. The experiments were conducted for 2 months in a shaking incubator at 60 rpm/min. The samples were cleaned using deionized water and dried in a vacuum oven at 80 °C for 24 h. Weight-loss values were calculated by comparing the weight ratios of the samples before and after degradation, as per Equation (4):

Weight Loss (%) = (W_b_ − W_a_)/(W_b_) × 100 (4)

where W_b_ and W_a_ are the weights of the polyurethane films before and after enzymolysis.

The morphology changes of TPU films before and after enzymolysis were characterized by SEM (COXEM EM 30AX plus, Daejeon, South Korea).

Composted soil was used according to the ISO 20200:2023 [47] test standard. The polymer films tested were a square 2.5 cm × 2.5 cm in size and 100 ± 30 µm thick, and the polymer films were buried in the composted soil at a temperature of 58 ± 2 °C.

## 4. Conclusions

In summary, a series of aliphatic CO_2_-based TPUs were successfully synthesized via CO_2_-based polyols, HMDI, and BDO, which provides a sustainable route to the fixation of CO_2_. All the synthesized TPUs exhibit an amorphous state and have only one glass transition temperature, leading to high transmittance. The mechanical and thermodynamic properties of TPUs can be regulated by changing the content of hard segments and the composition of soft segments. The incorporating of 10–30% PPG as the soft segment endows the TPUs with remarkable shape memory and self-healing characteristics, higher toughness, and thermal decomposition temperature as compared with PPCDL-TPU. Moreover, the CO_2_-based TPUs not only have impressive biocompatibility and water vapor-permeation resistance but also possess a certain biodegradability, foreshadowing a wide range of application prospects as an environmentally friendly polyurethane.

## Figures and Tables

**Table 1:** Preparation of TPUs with PPCDL and PPG.

Samples	Ingredient				*M*_n_ ^c^ (kg/mol)
	PPCDL ^a^ (g)	PPG ^b^ (g)	HMDI (g)	BDO (g)	
TPU-15%HS	7.3	0	1.2	0.057	34
TPU-25%HS	7.5	0	2.1	0.36	38
TPU-10%PPG-25%HS	6.4	0.64	2.0	0.32	39
TPU-20%PPG-25%HS	7.6	1.5	2.6	0.39	36
TPU-30%PPG-25%HS	7.8	2.3	2.9	0.41	41

^a^ OH value 62.3 mg/g. ^b^ OH value 112 mg/g. ^c^ NCO/OH = 1, content of hard segment = [(mass of HMDI + mass of BDO)/(mass of HMDI + mass of BDO + mass of PPG + mass of PPCDL)] × 100, Determined by GPC analyses (Figure S1).

**Table 2:** Characteristics of TPUs with with PPCDL and PPG.

Sample	*T*_g_ ^a^ (°C)	*T_d_*_5_ ^b^ (°C)	*T_d_*_50_ ^b^ (°C)	σ (MPa)	ε (%)	Hardness (Shore A)
TPU-15%HS	43	226	254	12.1 ± 0.6	384 ± 15	68
TPU-25%HS	32	222	248	14.7 ± 1.0	98.5 ± 12	75
TPU-10% PPG-25%HS	29	231	263	10.1 ± 0.5	438 ± 40	60
TPU-20% PPG-25%HS	26	225	265	7.5 ± 0.5	730 ± 35	55
TPU-30% PPG-25%HS	16	230	296	4.5 ± 0.4	886 ± 40	48

^a^ Determined at the second heating run by DSC. ^b^ Determined by TGA, T*_d_*_5_ = 5% weight-loss temperature, T*_d_*_50_ = 50% weight-loss temperature.

**Table 3:** Rf and Rr from thermomechanical testing.

Samples	1st Cycle		2nd Cycle		3nd Cycle		4th Cycle	
	R_f_	R_r_	R_f_	R_r_	R_f_	R_r_	R_f_	R_r_
TPU-10% PPG-25%HS	98.2	72.4	97.3	60.4	96.4	90.8	97.0	91.1
TPU-30% PPG-25%HS	99.8	73.8	98.8	92.1	98.5	93.6	99.5	93.5

**Table 4:** Molecular weight and mass of TPU following enzymatic hydrolysis by PBS + CALB for 66 days at 45 °C.

Sample	Weight Loss (wt%)	*M*_n_ (g/mol)	*M*_w_ (g/mol)	PDI
TPU-25%HS	original	38,000	67,000	1.76
	23.6	8500	15,000	1.76
TPU-20%PPG-25%HS	original	36,000	71,000	1.82
	15.9	10,300	20,000	1.94

## Data Availability

Data are contained within the article.

## Supplementary Materials

The following supporting information can be downloaded at: https://www.mdpi.com/article/10.3390/molecules29184364/s1, Figure S1. (a) ^1^H NMR spectrum, (b) GPC trace of PPCDL, (c) MALDI-TOF mass spectrum of PPCDL. A = −[OCH_2_CH(CH_3_)OCO]−; B = −[OCH_2_CH(CH_3_)]−; and C = −[OCH_2_CH_2_CH_2_CH_2_]−; Figure S2. GPC of TPU films; Figure S3. ^1^H NMR (500 MHz, DMSO-d6), TPU-20% PPG-25%HS films; Figure S4. DSC of PPCDL; Figure S5. XRD patterns of TPU films; Figure S6. Deformation of TPU-20% PPG-25%HS film during tensile process; Figure S7. Shape memory performance photos of TPU-10% PPG-25%HS; Figure S8. SEM micrographs of the surfaces for samples before (a), (b) and after (c), (d) enzymatic degradation in PBS buffer solution for 66 days; Table S1. Water vapor transmission rate (WVTR) for TPU films; Table S2. Molecular weight of TPU before and after composting degradation for 12 weeks; Figure S9. GPC of TPUs before and after composting degradation.
