# Supplementary material for: Self-Healable, Transparent, Biodegradable, and Shape Memorable Polyurethanes Derived from Carbon Dioxide-Based Diols
Source: Molecules. 2024 Sep 13;29(18):4364. doi: 10.3390/molecules29184364 (PMC11434357; doi:10.3390/molecules29184364)
Supplement: Supplementary file 1 [file molecules-29-04364-s001.zip › molecules-3164927-supplementary.pdf]

Supplementary Materials

Self-Healable, Transparent, Biodegradable, and Shape Memorable Polyurethanes Derived from Carbon Dioxide-Based Diols

Xin Huang [1], TingTing Zhao [1], ShuanJin Wang [1], Dongmei Han [2], Sheng Huang [1], Hui Guo [2], Min Xiao [1,*] and Yuezhong Meng [1,2,3,4,*]

[1] The Key Laboratory of Low-Carbon Chemistry & Energy Conservation of Guangdong Province, State Key Laboratory of Optoelectronic Materials and Technologies, School of Materials Science and Engineering, Sun Yat-sen University, Guangzhou 510275, China; 13890773752@163.com (T.Z.)
[2] School of Chemical Engineering and Technology, Sun Yat-sen University, Zhuhai 519000, China; handongm@mail.sysu.edu.cn (D.H.)
[3] Institute of Chemistry, Henan Provincial Academy of Sciences, Zhengzhou 450000, China
[4] College of Chemistry, Zhengzhou University, Zhengzhou 450001, China
* Correspondence: stsxm@mail.sysu.edu.cn (M.X.); mengyzh@mail.sysu.edu.cn (Y.M.)

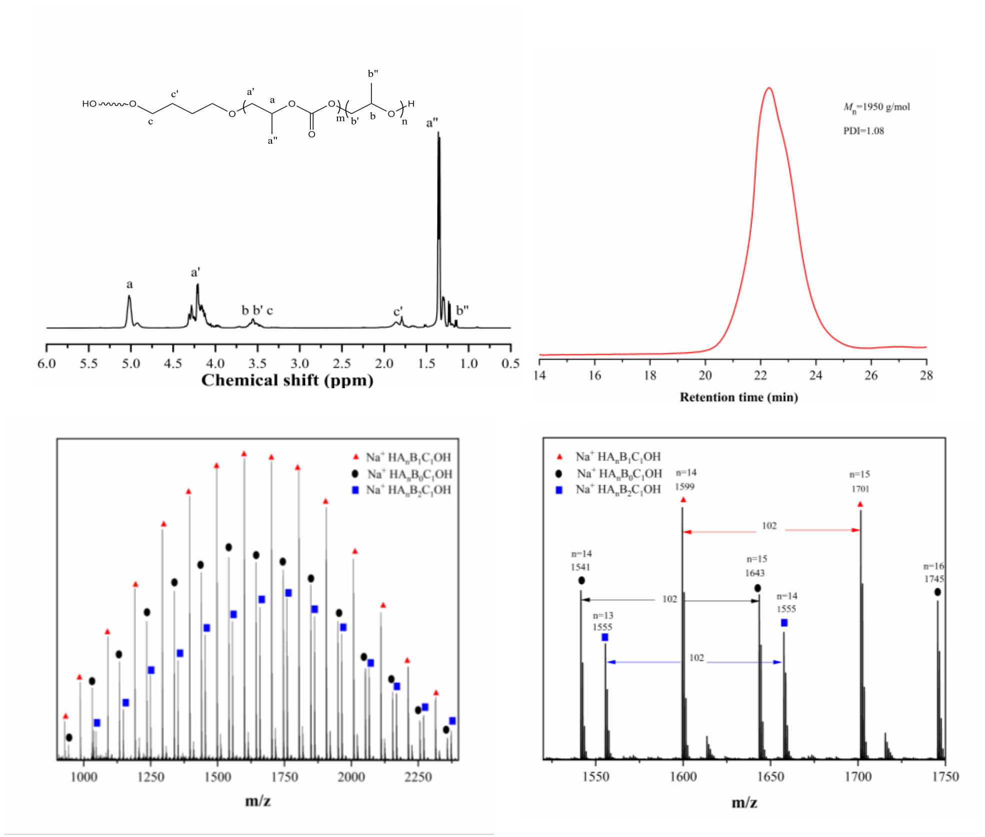

Figure S1. (**a**) ^{1}H NMR spectrum, (**b**) GPC trace of PPCDL, (**c**) MALDI-TOF mass spectrum of PPCDL. A =−[$OCH_2CH(CH_3)OCO$]−;B= −[$OCH_2CH(CH_3)$]−; and C =−[$OCH_2CH_2CH_2CH_2$]−;.(**d**) Zoom.

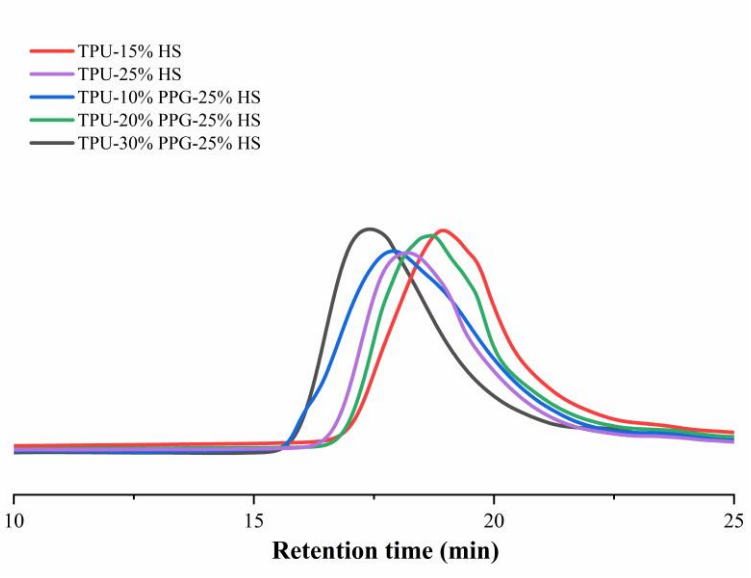

Figure S2. GPC of TPU films.

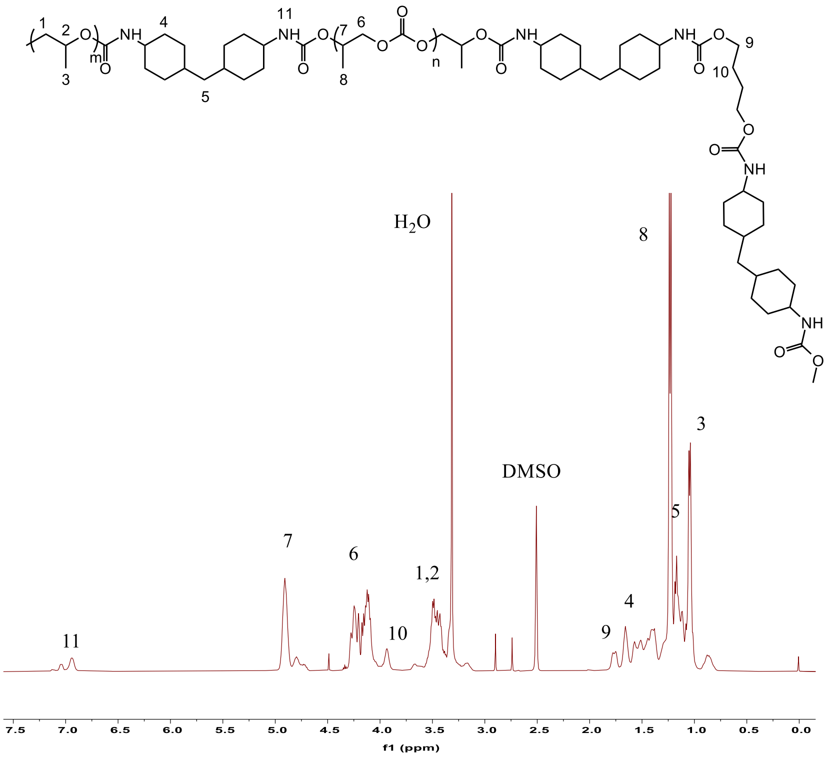

Figure S3. ^{1}H NMR (500 MHz, DMSO-d6),TPU-20% PPG -25%HS films.

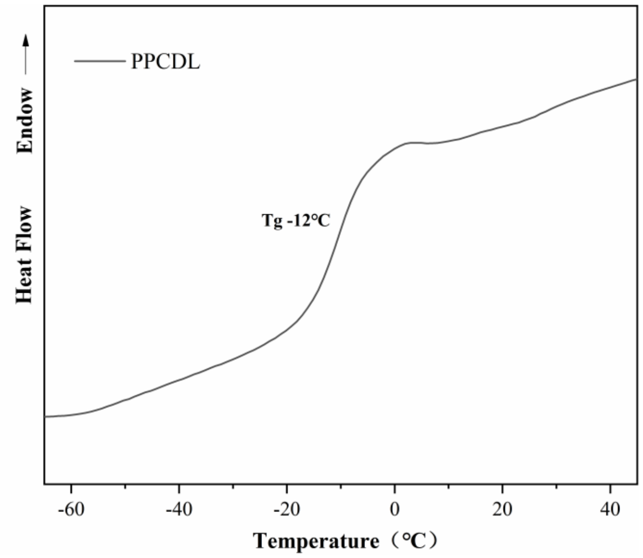

Figure S4. DSC of PPCDL.

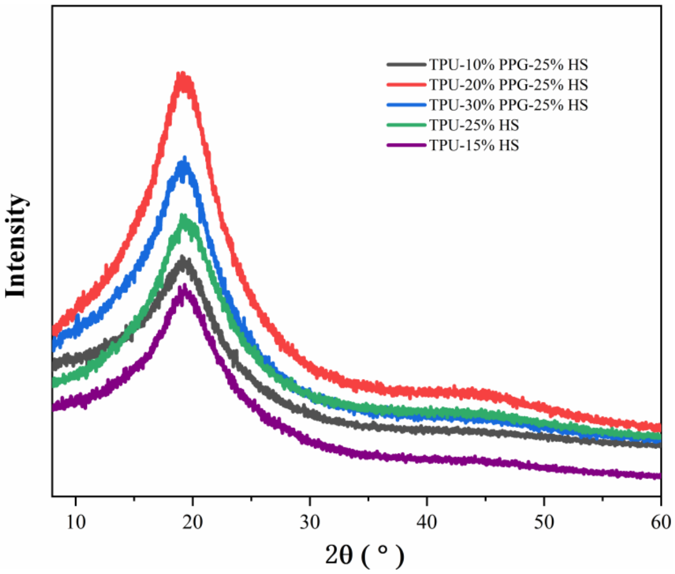

Figure S5. XRD patterns of TPU films.

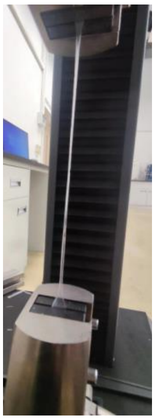

Figure S6. Deformation of TPU-20% PPG -25%HS film during tensile process.

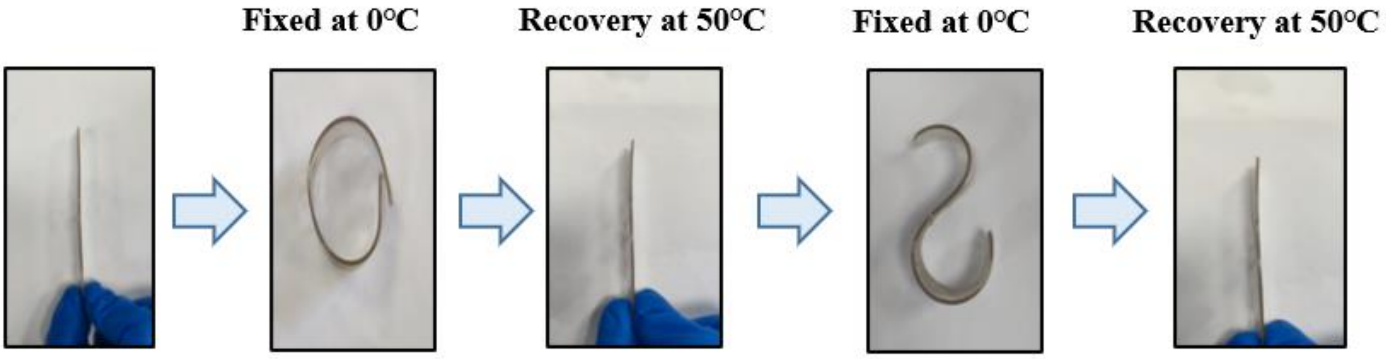

Figure S7. Shape memory performance photos of TPU-10% PPG -25%HS.

Table S1. Water vapor transmission rate (WVTR) for TPU films.

Sample	Thickness (μm)	Vapour transmission rate (WVTR) (g·m^{-2}·day^{-1})	Vapour transmission coefficient (WVP) (g·μm·m^{-2}·day^{-1})
TPU-15%HS	148	1.20	178
TPU-25%HS	160	1.07	171
TPU-10% PPG-25%HS	151	1.21	183
TPU-20% PPG-25%HS	195	1.12	219
TPU-30% PPG-25%HS	148	2.01	299

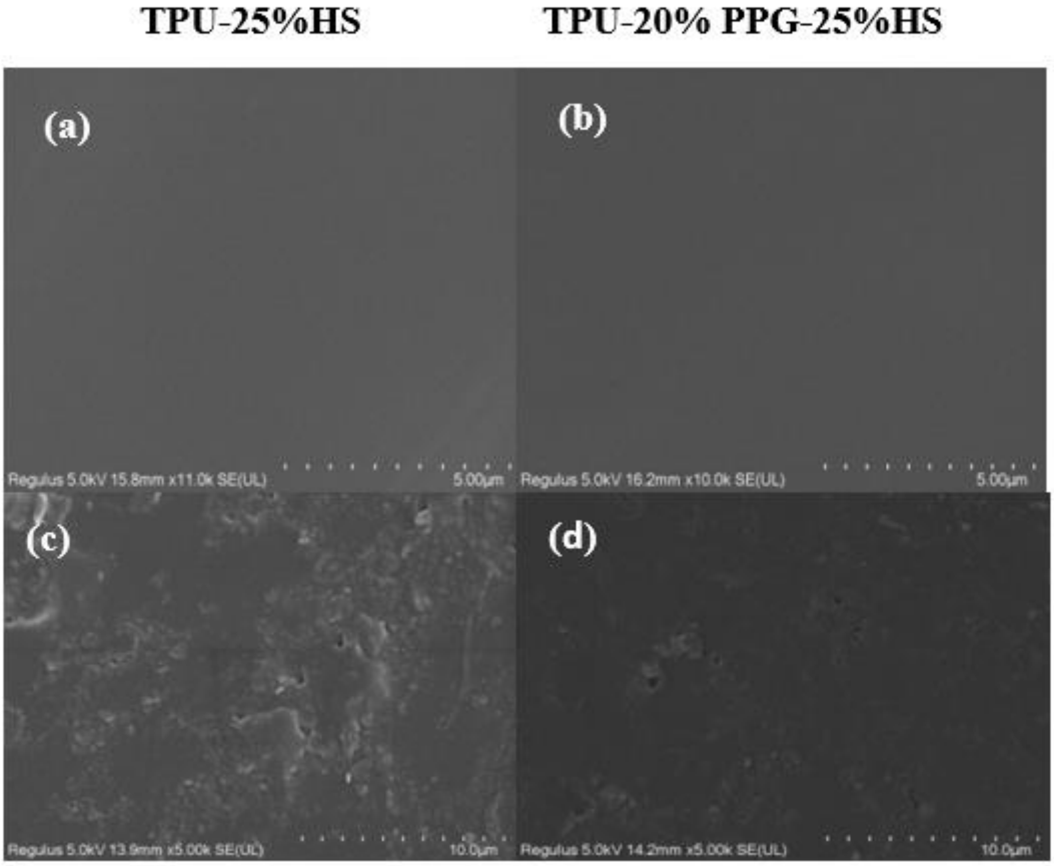

Figure S8. SEM micrographs of the surfaces for samples before (a), (b) and after (c), (d) enzymatic degradation in PBS buffer solution for 66 days.

Table S2. Molecular weight of TPU before and after composting degradation for 12 weeks.

Sample	M_n (g/mol)	M_w (g/mol)	PDI
TPU-25%HS	38000	67000	1.76
	9900	17000	1.72
TPU -20%PPG-25%HS	36000	71000	1.82
	28000	47300	1.69

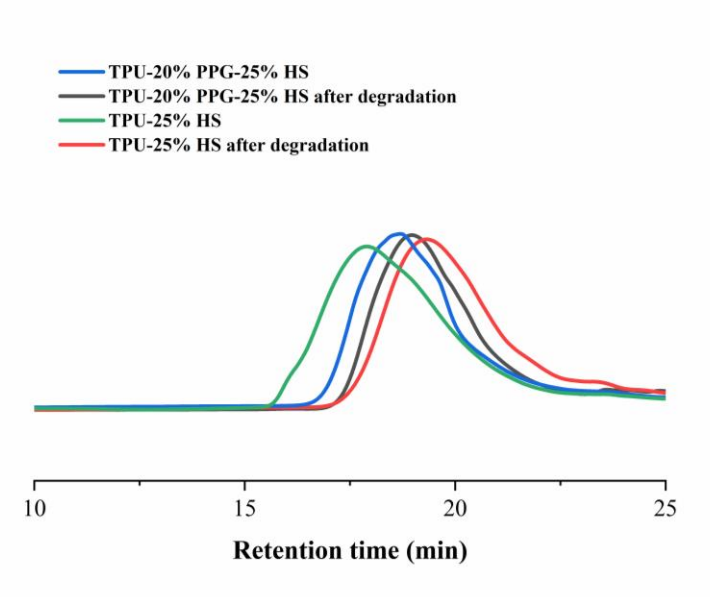

Figure S9. GPC of TPUs before and after composting degradation.
